# Rapid Analytical Method for Quantification of Gamma‐Hydroxybutyrate (GHB) in Hair by UPLC‐MS/MS

**DOI:** 10.1002/dta.3798

**Published:** 2024-09-15

**Authors:** Miriam Blanco‐Ces, Ana de‐Castro‐Rios, Angela Lopez‐Rabuñal, Maria Cobo‐Golpe, Angelines Cruz, Elena Lendoiro

**Affiliations:** ^1^ Toxicology Service Institute of Forensic Sciences, University of Santiago de Compostela Santiago de Compostela Spain

**Keywords:** alternative matrix, drug‐facilitated crime, GHB, hair, UPLC‐MS/MS

## Abstract

Gamma‐hydroxybutyrate (GHB), an endogenous compound related to the neurotransmitter gamma‐aminobutyric acid (GABA), is used as a therapeutic and recreational drug and as a “weapon” in drug‐facilitated crimes. The very short window of detection of GHB in conventional matrices (blood and urine) makes necessary the use of alternative matrices like hair. Hair has a long window of detection and the possibility to perform segmental analysis, which makes it very useful for proving GHB intake. In the present work, a method for quantification of GHB in hair was developed and validated. Hair (10 mg) was washed twice with dichloromethane and then incubated at room temperature with Milli‐Q water in an ultrasound bath for 30 min. Analysis was performed by UPLC‐MS/MS using a CORTECS UPLC HILIC (1.6 μm), 2.1 × 100‐mm column, and a gradient with acetonitrile and ammonium acetate (10 mM) at pH 6.0, with a total run‐time of 10 min. For detection, a triple quadrupole mass spectrometer in ESI negative mode was used. The method was validated, following the criteria established in the “AAFS Standard Practices for Method Validation in Forensic Toxicology” guideline, obtaining satisfactory results for linearity (0.5–50 ng/mg), accuracy (95.0%–103.2%), imprecision (< 10.2%), limit of detection (0.1 ng/mg) and quantification (0.5 ng/mg), exogenous selectivity (no interferences), matrix effect (less than −44.2%), extraction efficiency (> 86.4%), process efficiency (> 46.1%), and autosampler stability (< 4.3%). The method was used for the analysis of 26 authentic hair samples, 25 from non–drug users, obtaining values between < LOQ and 6.25 ng/mg of endogenous GHB and 1 from a former GHB chronic user to prove abstinence.

## Graphical abstract

A rapid and simple UPLC‐MS/MS method for quantification of GHB in 10 mg of hair was developed. The method was satisfactorily validated, showing a linear range of 0.5–50 ng/mg, which allows us to quantify endogenous and exogenous GHB concentrations.

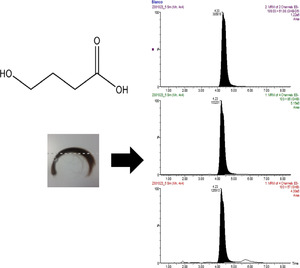

## 1 Introduction

Gamma‐hydroxybutyrate (GHB) is an endogenous compound that acts as a central nervous system depressant with dual functions as a precursor and a metabolite of the neurotransmitter gamma‐aminobutyric acid (GABA) [1]. However, GHB is also administered as a xenobiotic because of its sedative and euphoric effects. On the one hand, it has been used as a recreational drug of abuse. On the other hand, this compound has been approved as a therapeutic drug under the form of sodium oxybate for the treatment of narcolepsy, the treatment of alcohol withdrawal syndrome, and the maintenance of alcohol abstinence [2].

The effects and pharmacological characteristics of GHB make it an “ideal” agent for its use in drug‐facilitated crimes. GHB induces drowsiness, sedation, amnesia, and victims' inability to resist the aggression. Moreover, it is soluble in water and is rapidly absorbed after oral administration, with a peak plasma concentration in 25–45 min, and its half‐life in plasma is 30–50 min [1, 3, 4, 5].

Analytical detection of GHB presents several pitfalls. First, it is rapidly eliminated from the body. When a detection limit of 1 mg/L is set, the detection window in serum is estimated in 4–5 h and in urine 8–10 h [6]. Therefore, its detection through conventional matrices such as blood or urine is difficult. Keratinized matrices, such as hair, can be an alternative for GHB analysis. Hair stably incorporates xenobiotics to their structure by passive diffusion from blood, which allows a long‐term detection window (limited only by hair length) and the possibility of establishing the chronological profile of consumption by segmental analysis. Another pitfall of analytical detection of GHB is its presence as an endogenous compound, which complicates its quantification in biological samples, as detected concentrations may include contributions from both endogenous and exogenous sources [1]. Segmental analysis of hair samples can assist in the differentiation between endogenous and exogenous exposure. An approach based on the calculation of a ratio between GHB concentrations in different hair segments of the same individual, corresponding with a period of nonexposure to exogenous GHB (baseline concentration) and a period of alleged exposure, has been used by several authors [7, 8, 9, 10]. Finally, GHB is a very polar small molecule (104.1 g/mol) with weak ionization efficiency, which represents another difficulty for its analysis [7]. The use of gas chromatography–mass spectrometry (GC–MS) is more common [7, 8, 11, 12, 13, 14, 15, 16, 17, 18, 19, 20, 21, 22, 23, 24, 25, 26], although it is necessary to implement a derivatization process as an additional preanalytical step, increasing time and cost of the analysis. The use of liquid chromatography–tandem mass spectrometry (LC–MS/MS) avoids this step but requires a more sensitive equipment.

The aim of the present work is to develop and validate a rapid method for the detection of GHB in hair by ultra‐performance liquid chromatography coupled to tandem mass spectrometry (UPLC‐MS/MS), which allows the quantification of GHB in multiple hair segments simultaneously and in a short time.

## 2 Material and Methods

### 2.1 Chemicals, Reagents, and Materials

GHB and GHB‐d_6_, both at 1.0 mg/mL in methanol, were purchased from Cerilliant (Round Rock, Texas, United States). Water was purified with a Milli‐Q water system (Millipore, Le‐Mont‐sur‐Lausanne, Switzerland). Reagent‐grade acetonitrile and methanol were from Fisher Chemical (Loughborough, United Kingdom). Reagent‐grade glacial acetic acid was from Scharlab (Sentmenat, Spain). Reagent‐grade dichloromethane and ammonium acetate were purchased from VWR Chemicals (Pennsylvania, United States).

### 2.2 Hair Samples

Nonuser hair samples were obtained from healthy volunteers and were employed for the preparation of calibrators and quality control (QC) samples.

Authentic hair samples (*n* = 26) were collected at the Institute of Forensic Sciences in Santiago de Compostela, Spain. These authentic cases were anonymized forensic cases submitted to this institution. Twenty‐five cases were from non–drug users to determine endogenous GHB concentrations, and one case was from a chronic GHB user to determine cessation of the consumption.

All samples were stored at room temperature in their original packing (aluminum foil or envelope) until analysis.

### 2.3 Preparation of Calibration and QC Solutions

A stock solution at 10 μg/mL in methanol was prepared from GHB standard at 1.0 mg/mL. Further dilutions in methanol were prepared at 1.0 and 0.1 μg/mL. An 8‐point calibration curve from 0.5 to 50 ng/mg was generated by the addition of 25–100 μL of the appropriate working solution to 10 mg of nonuser hair.

Independent stock and working solutions at 0.3, 0.8, and 8.0 μg/mL were prepared for the generation of low, medium, and high QC samples (1.5, 4, and 40 ng/mg, respectively).

An internal standard (IStd) solution at 1 μg/mL was prepared by dilution of the original IStd standard solution in methanol.

### 2.4 Sample Preanalytical Procedure

For decontamination, hair samples were placed into 40‐mL Pyrex tubes with lids and washed with 5 mL of dichloromethane for 2 min twice. The dichloromethane was decanted, and the tubes containing the hair samples were placed into the oven for 5 min at 40°C. The last wash was evaporated to dryness for a posterior analysis if necessary.

A 10‐mg aliquot of hair was placed into a Precellys tube containing metal beads and pulverized in the Precellys homogenizer using the following settings: 6500 vibrations per minute, 3 cycles of 60 s, of which 46 s is of agitation and the remaining 14 s is of rest. The resulting powder was transferred to 2‐mL propylene microtubes. After that, two consecutive aliquots of Milli‐Q water (0.5 mL each) were added to the Precellys tube to completely transfer the hair powder to the propylene microtube.

For incubation, 50 μL of the IStd at 1 μg/mL was added to the propylene microtube and placed in an ultrasound bath at room temperature for 30 min. After centrifugation at 14,500 rpm for 15 min, the supernatant was evaporated to dryness using a N_2_ evaporator at 37°C. The dried extract was reconstituted in 50 μL of acetronile:methanol (3:1, v/v) and centrifuged at 14,500 rpm for 5 min. The resultant supernatant was transferred into UPLC vials, and 5 μL was directly injected into the UPLC‐MS/MS system.

### 2.5 Instrumentation

An ACQUITY UPLC H‐Class with a quaternary solvent manager (QSM) pump (Waters Corp., Milford, MA, United States) was used for the chromatographic separation. Chromatography was performed using a CORTECS UPLC hydrophilic interaction liquid chromatography (HILIC) (2.1 × 100 mm, 1.6 μm) analytical column (Waters Corp.), maintained at 40°C. The composition of the mobile phase was 10 mM of ammonium acetate at pH 6.0 in water (A) and acetonitrile (B), at a flow rate of 0.3 mL/min. The chromatographic gradient was programmed as follows: 5% of A for 3 min; then A linearly increased from 5% to 10% until minute 3.8 and from 10% to 20% until minute 5.0; after that, A increased to 60% until minute 6.0; and finally, A returned to initial conditions at minute 6.5 and equilibrate until minute 10. The autosampler temperature was kept at 6°C.

A Xevo TQ‐XS triple quadrupole mass spectrometer (Waters Corp.) was employed for detection. The instrument was operated in electrospray in negative mode (ESI−) with the following optimized settings: capillary voltage of 2.75 kV; source block and desolvation gas (nitrogen) temperatures of 150°C and 250°C, respectively; and desolvation and cone gas (nitrogen) flow rates of 600 and 150 L/h, respectively.

Data were recorded on multiple reaction monitoring (MRM) mode. Individual infusions of solutions at 10 μg/mL of GHB and its IStd were performed at a 10 μL/min flow rate to select MRM transitions, cone voltages (CVol), and collision energies (ce) for both analytes. Two transitions were monitored for GHB (103 > 85, CVol: 18 V, ce: 8 eV as quantified and 103 > 57, CVol: 18 V, ce: 12 eV as qualified) and one transition for GHB‐d_6_ (109.03 > 61.08, CVol: 20 V, ce: 12 eV).

Data acquisition was controlled with MassLynx V4.2 SCN 1035 software and processed with TargetLynx V4.2 SCN 1035.

### 2.6 Method Validation

Validation was based on the guide “Standard Practices for Method Validation in Forensic Toxicology” from the American Academy of Forensic Sciences (AAFS) [27]. The following parameters were studied for method validation: linearity, limits of detection (LODs) and quantification (LOQs), imprecision and accuracy, carryover, selectivity, matrix effect, recovery, total efficiency of the process, and autosampler stability (72 h).

**FIGURE 1:**
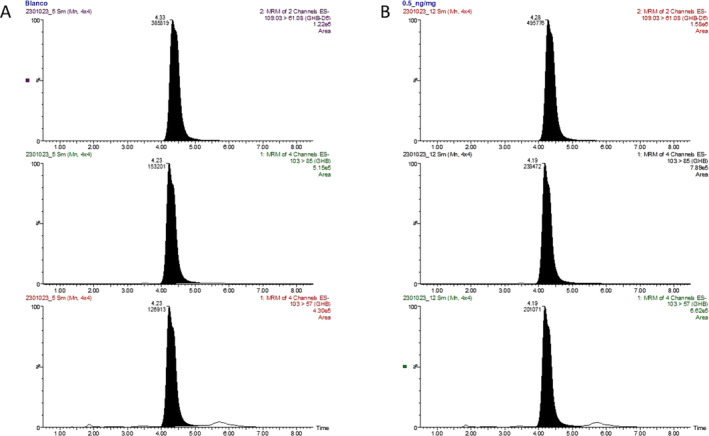
MRM chromatogram of GHB and its IStd (GHB‐d_6_) in the same hair sample corresponding to endogenous (A) and fortified at 0.5 ng/mg (B) concentration.

The “standard addition method” was used to perform the validation procedure due to the endogenous presence of GHB in authentic hair samples (Figure 1A). This method involves adding a known amount of GHB to the hair sample used for preparing the calibrators and QCs, which has a baseline concentration of endogenous GHB, then determining the baseline amount of GHB (area under the curve) in this hair sample, and subtracting this value to all calibrators and QCs. Once all the recalculated response values have been obtained, the recalculated calibration curve was determined and used to quantify QC samples. The same procedure was used to quantify concentrations in real hair samples.

The best calibration model was evaluated by the generation of five calibration curves from 0.5 to 50 ng/mg, analyzed on five different days. Acceptance criteria included a coefficient of determination (*r* ^2^) ≥ 0.99 and residuals ≤ 20% at each concentration level.

The LOD was defined as the lowest concentration at which the two MRM transitions monitored for GHB could be identified with a signal‐to‐noise ratio of ≥ 3 and an appropriate ion ratio. LOD was determined by the analysis of three replicates of fortified hair samples at decreasing concentrations (0.25 and 0.1 ng/mg).

The LOQ was defined as the lowest concentration that could be quantified with adequate precision (%CV ≤ 20%) and accuracy (percentage of target concentration ± 20%). The LOQ was calculated by the analysis of three replicates at the lowest concentration of the calibration curve (0.5 ng/mg).

Imprecision and accuracy in fortified hair samples were evaluated at low, medium, and high QC concentrations (1.5, 4.0, and 40 ng/mg, respectively) by the analysis of three replicated at each concentration level on five different days (*n* = 15). Imprecision was evaluated by the calculation of the coefficient of variation (%CV). Accuracy was expressed as the percentage of the nominal concentration and was required to be within 80%–120% of the target concentration.

To measure carryover, a blank matrix sample was injected immediately after the highest calibrator over 5 days. Carryover was dismissed if the signal obtained did not exceed 10% of the signal of the lowest calibrator.

Selectivity was evaluated by assessment of exogenous interferences. Endogenous interferences were not evaluated because GHB is an endogenous compound, so levels of GHB were detected in all hair samples. Exogenous interferences were assessed by the analysis of nonuser hair samples fortified with common drugs of abuse and medicines at 50 ng/mg (highest concentration of the calibration curve). The following drugs were tested: morphine, codeine, 6‐acetylmorphine, methadone, 2‐ethylidene‐1,5‐dimethyl‐3,3‐diphenylpyrrolidine (EDDP), amphetamine, methamphetamine, 3,4‐methylenedioxyamphetamine (MDA), 3,4‐methylenedioxymethamphetamine (MDMA), 3,4‐methylendioxyethylamphetamine (MDEA), cocaine, benzoylecgonine, ecgonine methyl ester, cocaethylene, nicotine, cotinine, fentanyl, amitriptyline, paroxetine, omeprazole, paracetamol, ibuprofen, diclofenac, naproxen, alprazolam, temazepam, lormetazepam, lorazepam, flunitrazepam, 7‐aminoflunitrazepam, clonazepam, diazepam, nordiazepam, oxazepam, triazolam, nitrazepam, bromazepam, zolpidem, and zopiclone. Acceptance criteria are that the area detected for the analyte should be equal to the one from the blank sample and/or three times inferior to the LOD area.

Matrix effect, the recovery, and the total efficiency of the process were evaluated at low and high QC concentrations using hair samples from 10 different non–GHB users. The matrix effect was assessed by comparing the analytes' peak area in blank samples fortified with GHB and its IStd after sample preparation with peak area when the analytes were prepared in acetonitrile:methanol (3:1, v/v). Recovery was calculated by comparing the analytes' peak area in samples fortified before extraction with those observed in samples fortified after extraction. Total process efficiency was determined by comparing the analytes' peak area in samples fortified before extraction with peak area when the analytes were prepared in acetonitrile:methanol (3:1, v/v). The analyte area in the blank samples was subtracted to the analyte area in the corresponding samples fortified before and after extraction.

Hair samples are stabled at room temperature and do not require special storage conditions, so the stability of the extracts in the autosampler at 6°C after 24 and 72 h of storage was exclusively evaluated. The stability of the analyte in hair extracts was evaluated at low, medium, and high QC concentrations using three replicates per concentration level. QC samples were analyzed fresh and reinjected into the UPLC‐MS/MS system after storage in the autosampler. The percentage of analyte loss with respect to the value obtained in the fresh QC samples was calculated. The stability of the extracts was considered acceptable when the loss percentage in the reinjected QC samples was ≤ 20%.

### 2.7 Application to Real Specimens

In order to evaluate the performance of the method, hair samples from 25 non–drug users were analyzed to detect endogenous levels of GHB. The quantification was carried out as indicated above, using the “standard addition method.” For 24 cases, a single segment was analyzed, but for one case, a segmental analysis was performed (36 segments of 0.5 cm).

Moreover, one case from a chronic GHB user was analyzed to determine cessation of GHB consumption. Two samples were collected from the same individual, one just after cessation of active chronic consumption of GHB (3 cm) and one after 3 months of abstinence (2 cm). Both samples were segmented in 1‐cm segments.

## 3 Results

### 3.1 Method Development

During the development of this method, different parameters from the sample treatment process were studied and optimized. First, the impact of grinding was tested to evaluate the total loss of hair powder and incubation solvent during sample treatment. The percentage of total loss was 4.1%–7.2% (*n* = 3), showing a nonsignificant loss. For the incubation, Milli‐Q water, MeOH, ammonium formate of 1 mM, and a mixture of methanol:acetonitrile:ammonium formate of 1 mM (25:25:50; v:v:v) were evaluated as solvents. The highest recoveries were obtained with water. Moreover, different incubation temperatures and the use of sonication were also evaluated to improve GHB recoveries; the best results were obtained when sonicating for 30 min at room temperature.

Additionally, two types of extraction techniques were tested: liquid–liquid extraction with Milli‐Q water, sodium hydroxide of 1 M, methanol, or acetonitrile:hydrochloric acid of 0.1 M (50:50, v:v) with ethyl acetate and solid‐phase extraction (SPE) with Water OASIS MAX cartridges, and the results were compared with those achieved when no extraction was performed. Cleaner extracts were obtained with the SPE approach compared with the liquid–liquid extraction. However, the simple and rapid dilution of the sample during incubation showed better recovery with a sufficient signal‐to‐noise ratio for the purpose of the method.

Regarding the chromatography separation, different columns, mobile phases, and gradients were evaluated. The use of reverse‐phase columns (ACQUITY UPLC HSS T3 1.8 μm and ACQUITY UPLC BEH C18 1.7 μm) did not provide GHB retention and produced less sensitivity because of the coelution with matrix interferences. The high polarity of GHB requires the use of analytical columns with adequate retention for this type of compound such as the selected HILIC column. The best results were obtained using a gradient with acetonitrile and ammonium acetate (10 mM) at pH 6 as the mobile phase. The pH of the ammonium acetate solution was a critical point to obtain a good chromatographic separation, because it allowed separation of the GHB qualitative MRM transition from an impurity present in the sample that eluted at a close retention time.

### 3.2 Validation

The calibration range was 0.5–50 ng/mg, fulfilling the acceptance criteria for the coefficient of determination (*r* ^2^ ≥ 0.99) and the residuals (< 20%) using a linear, nonforced, and 1/*x* weighting model.

The LOD was established at 0.1 ng/mg. On the other hand, the LOQ achieved in this method was established at 0.5 ng/mg (Figure 1B), obtaining an accuracy of 91.9% and an imprecision of 12.7% and therefore fulfilling an adequate sensitivity to quantify endogenous and exogenous levels of GHB.

Accuracy ranged between 95.0% and 103.2% of the theoretical value, which is within the acceptance criteria. Likewise, the between‐day, within‐day, and total imprecision values were between 4.2% and 10.2% (Table 1).

**TABLE 1:** Results for imprecision (intraday, interday, and total) and accuracy for GHB at three concentration levels (low, medium, and high QC).

	Imprecision (%CV) (*n* = 15)		Accuracy (% of the theoretical value) (*n* = 15)	
Concentration	Intraday	Interday	Total	Accuracy
Low QC (1.5 ng/mg)	5.4	8.1	9.7	95.0
Medium QC (4.0 ng/mg)	9.1	4.7	10.2	103.2
High QC (40 ng/mg)	4.2	7.3	8.4	97.4

The developed method was selective regarding the detection of exogenous interferences, because the response obtained for GHB after fortifying a hair sample from a non–GHB user with common drugs of abuse and pharmaceuticals was not different from that observed in the sample without making this addition.

With regard to the matrix effect, ion suppression was detected, for both GHB (−44.2 and −36.0, at the low and the high QC concentrations, respectively) and its IStd (−60.1 and −47.6, at the low and the high QC concentrations, respectively) (Table 2). The effect of ion suppression could be compensated by the addition of GHB‐d_6_, which suffers the same matrix effect. Recovery of GHB was ≥ 86.4%, with a total process efficiency ranging between 35.9% and 53.0% because of the ion suppression (Table 2).

**TABLE 2:** Results obtained for the matrix effect, recovery, and process efficiency at low and high QC levels.

Compound	Concentration	Matrix effect (%CV, *n* = 10)	Recovery (%CV, *n* = 10)	Process efficiency (*n* = 10)
GHB	Low QC (1.5 ng/mg)	−44.2 (−44.3)	87.1 (39.1)	46.1
	High QC (40 ng/mg)	−36.0 (−39.9)	86.4 (25.5)	53.0
GHB‐d_6_	Low QC (1.5 ng/mg)	−60.1 (−15.5)	91.5 (17.3)	35.9
	High QC (40 ng/mg)	−47.6 (−20.3)	98.7 (20.6)	50.6

No carryover was detected in the injection immediately after the highest calibrator. In addition, GHB proved to be stable after 72 h in the autosampler at 6°C, with a percentage loss of < 4.3% in all cases.

### 3.3 Application to Real Specimens

The method proved its performance by the analysis of 24 real hair specimens from non–drug users to determine endogenous GHB concentrations in a single segment. Table 3 shows the concentrations obtained, with values between < LOQ and 6.25 ng/mg. Moreover, a segmental analysis was performed in one real hair specimen from a non–drug user to determine the chronological pattern of endogenous GHB concentrations (Table 3), ranging between < LOQ and 1.96 ng/mg.

**TABLE 3:** Endogenous GHB concentrations in hair samples from 25 different individuals, all of them non–drug users.

One‐segment samples (*n* = 24)		Segmental analysis (*n* = 1)		
Sample	Endogenous GHB concentration (ng/mg)	Sample	Segment (0.5 cm)	Endogenous GHB concentration (ng/mg)
1	0.84	25	1	< LOQ
2	1.94		2	0.58
3	1.25		3	1.96
4	3.03		4	0.69
5	1.88		5	0.70
6	3.83		6	0.71
7	3.59		7	0.85
8	2.11		8	0.64
9	2.50		9	0.90
10	1.21		10	0.93
11	3.29		11	< LOQ
12	< LOQ		12	0.79
13	< LOQ		13	0.51
14	0.61		14	0.62
15	6.25		15	0.73
16	0.60		16	0.55
17	< LOQ		17	0.58
18	< LOQ		18	< LOQ
19	1.92		19	< LOQ
20	1.30		20	< LOQ
21	< LOQ		21	< LOQ
22	< LOQ		22	< LOQ
23	0.85		23	< LOQ
24	1.19		24	< LOQ
			25	< LOQ
			26	< LOQ
			27	< LOQ
			28	< LOQ
			29	< LOQ
			30	< LOQ
			31	< LOQ
			32	< LOQ
			33	< LOQ
			34	< LOQ
			35	< LOQ
			36	< LOQ

In addition, two samples from a chronic GHB user were analyzed to prove cessation of consumption. The first sample, corresponding to the period with active consumption of GHB, was divided into three segments of 1 cm and showed the following concentrations: 0.79 ng/mg (Segment 1, proximal), 0.86 ng/mg (Segment 2), and 1.04 (Segment 3, distal). The second sample, collected after 3 months of abstinence, was divided into two segments of 1 cm and showed the following concentrations: 0.38 ng/mg (Segment 1, proximal) and 0.50 ng/mg (Segment 2, distal).

## 4 Discussion

The developed method for the detection and quantification of GHB in hair showed some advantages over previously published methods. First, the use of LC–MS/MS allows us to avoid the time‐consuming derivatization step necessary in GC–MS methods [7, 8, 12, 13, 14, 15, 16, 17, 18, 19, 20, 21, 22, 23, 24, 25, 26]. Second, the amount of hair employed (10 mg) is lower than the majority of previous methods, the use of 20 [6, 13, 17, 19, 28], 25, or 50 mg of hair [22, 29, 30, 31, 32] being common. Although some methods use the same amount of hair (10 mg) [20, 33, 34], and other two use a lower amount (5 mg) [8, 26]. However, these methods included a more complicated and laborious sample pretreatment, such as derivatization [8, 20, 26], SPE extraction [20], liquid–liquid extraction [34], and/or longer incubation times [33]. The use of small amounts of hair to perform the GHB analysis is relevant because it is common to have an initial small amount of sample, and it is also necessary to segment the hair lock into very small segments (3–5 mm) to discriminate endogenous and exogenous levels. Therefore, even for those methods that use low amounts of hair (5–10 mg), it is a common limitation to lack enough hair samples to perform segmental analysis when some real cases are analyzed and small segments (3–5 mm) are used.

Moreover, in the present method, the extraction of GHB from the hair matrix and its purification are performed in a single step and using Milli‐Q water. The high polarity of the water molecule is ideal to dissolve and extract a highly polar compound such as GHB. Previous published methods have used water for GHB extraction from the hair matrix [13, 19], but they needed longer incubation times (Van Elsué et al. [19] used sonication with 2 mL of water for 90 min, and Paul et al. [13] sonicated the sample with water overnight) and, in addition, a purification step with SPE cartridges. In the present method, GHB extraction is carried out in 30 min by sonication, and it is not necessary to perform the subsequent SPE, obtaining a similar extraction recovery to that of previous methods (> 86% vs. 87% [19]). Reducing sample pretreatment decreases analysis time and reduces the risk of analyte loss.

Only Ramírez Fernández et al. [35] performed the extraction and purification of the GHB from the hair matrix in a single incubation step and with the same amount of sample (10 mg). However, instead of water, a mixture of acetonitrile:methanol:ammonium formate of 1 mM (25:25:50, v:v:v) was used. As mentioned, this solution was also tested, but better results were obtained with water, achieving similar the percentage of recovery in both methods (> 86% in this method vs. 88% in Ramírez Fernández et al. [35]) and avoiding the use of organic solvents.

Other previous methods reached similar recoveries (88.3% [32]) to, slightly higher (> 92.3% [30], 92% [33], 87% [19]) than, or slightly lower (> 71% [20]; > 61.5 [17]) than that obtained in the present method. Only the method of Martz et al. [34] obtained a recovery of 23%, which, as they explain, may be due to the type of extraction used (liquid–liquid). As hair is a solid matrix, in real samples, the percentage of recovery could be different because the compounds are incorporated from the body into the hair and not on its surface like when the samples are spiked. However, all these studies evaluated recovery following a similar protocol to that in the present work using spiked samples.

Our method proved to be linear from 0.5 to 50 ng/mg, being a wider range than that obtained by the methods of Bertol et al. [30] (0.5–30.0 ng/mg) and Chagas et al. [20] (0.6–16 ng/mg) and similar to Jagerdeo et al. [31] (0.4–50 ng/mg) and Busardò et al. [32] (0.5–100 ng/mg). Meng et al. [22] (0.03–10 ng/mg), Shi et al. [17] (0.05–15 ng/mg), Ramírez Fernández et al. [35] (0.06–25 ng/mg), Schröck et al. [6] (0.1–10 ng/mg), Pascali et al. [28] (0.1–50 ng/mg), and Stout et al. [29] (0.2–100 ng/mg) achieved a lower LOQ but using a larger amount of hair (20–50 mg). Only Ramírez Fernández et al. [35] was able to quantify 0.06 ng/mg using the same amount of hair. However, this lower LOQ did not improve the detection of positive cases.

The method developed in this work is suitable for the detection and quantification of GHB in hair both for endogenous levels and for exogenous administrations in cases of recreational use or drug‐facilitated crimes. In other previous studies, the endogenous levels obtained for nonuser individuals varied between 0.033 and 15.4 ng/mg (Table 4), which are in accordance with those obtained in this study (< LOQ–6.25 ng/mg) after the analysis of hair samples from 25 different individuals. Moreover, the segmental analysis of one of those samples showed the importance of dismissing the closest hair segments to the scalp to avoid possible sweat contamination, which can anomalously elevate endogenous concentrations [10]. In this case, the first 1.5 cm of the hair lock showed a maximum concentration of 1.96 ng/mg, whereas Segments 4–18 (corresponding with the next 6.5 cm) showed concentrations lower than 0.93 ng/mg (2.1 times lower than the proximal section). After that, all concentrations were < LOQ (Segments 19–36) probably because of the washout effect.

**TABLE 4:** Endogenous GHB levels reported in the literature.

Reference	Number and type of hair samples analyzed	Endogenous GHB levels (ng/mg)
Bertol et al. [7]	Males: 90; females: 15	Chest hair (only males): 0.205–1.511 Pubic hair: 0.353–1.913 (males); 0.310–1.244 (females)
Bertol et al. [30]	Black hair: 10; blond hair: 10; dyed hair: 10	Black hair: < LOD (0.3)–4.49; blond hair: 0.58–5.09; dyed hair: 0.61–4.02
Castro et al. [15]	32 postmortem cases with information on cause of death and autopsy data	0.16–3.12 (except four samples, all GHB concentrations were < 2.0 ng/mg)
Chagas et al. [20]	23 volunteers (21 adults; 2 children)	< LOQ (0.6)–1.5
Goullé et al. [26]	Black hair: 19; brown hair: 30; blond hair: 12	Black hair: 0.32–1.54; brown hair: 0.41–1.86; blond hair: 0.35–0.95
	12 samples for hair segmental analysis (3‐mm‐long segments)	0.31–8.4
Jagerdeo et al. [31]	20	< LOQ (1.2)–4.4
Kintz et al. [8]	Males: 8; females: 16	0.5–12.0
Martz et al. [34]	Males: 26; females: 62	Males' mean concentration: 0.935 ± 0.887; females' mean concentration: 0.673 ± 0.613
Meng et al. [22]	Males: 25; females: 25	Males: 0.35–4.17; females: 0.34–4.10
Mestria et al. [24]	Males: 10; females 10	Males: 0.1–10.2; females: 0.6–15.4
Pascali et al. [28]	60 adults; 5 children below 2 years old	0.033–0.96
Shi et al. [17]	Males: 35; females: 31	Males: 0.92–4.91; females: 0.28–1.95
Schröck et al. [6]	Males: 2	Head hair: 0.1–0.6 Beard hair: 0.2–3.5
	27 individuals	0.1–1.3 (in 8 hair samples, GHB concentration was below the LOQ, 0.1)
Vaiano et al. [16]	Males: 75; females: 75	0.274–2.839 Males' mean concentration: 0.829; females' mean concentration: 0.596
Van Elsué et al. [19]	Males: 12; females: 8	0.3–2.0
Wang et al. [33]	Red hair: 1; brown hair: 5; blond hair 2; black hair: 2	< LOQ (0.32)–0.99

Likewise, the concentrations found in previous different real cases with active GHB consumption were between 0.8 and 594 ng/mg (Table 5). The real case from a GHB abuser analyzed in this study showed concentrations for active consumption (0.79–1.04 ng/mg) in accordance with the lower limit of the range found in the literature. However, it was possible to establish the cessation of the consumption using the approach that each subject serves as his or her own control [7, 8, 9, 10] by the calculation of a ratio between GHB concentrations in a period of nonexposure and a period of alleged exposure. Following this criterion, the GHB concentrations in the period of abstinence were two times lower than the ones from the active period of consumption.

**TABLE 5:** GHB concentrations described in the literature after exogenous administration.

Reference	User data	Hair sample	GHB concentration (ng/mg)
Bertol et al. [14]	40‐year‐old man with a history of GHB use, but who has not used GHB for 2 months following house arrest	A 17‐cm lock 1‐cm segments for the first 3 cm (S1, S2, and S3) and into 2‐cm segments for the remaining 14 cm	S1: 4.08 S2: 1.26 S3: 1.38 S4: 4.56 S5: 6.48 S6: 7.23 S7: 8.56 S8: 7.39 S9: 8.95 S10: 9.15
Bertol et al. [30]	29‐year‐old male brought to the emergency department for an acute intoxication due to alcohol and GHB	First hair sample, 2.2 cm (1 day after intoxication) Second hair sample, 3 cm (32 days after intoxication) Third hair sample, 4.6 cm (84 days after intoxication) 5‐mm segments	First hair sample: S1: 2.7 S2: 1.5 S3: 1.39 S4: 1.52 Second hair sample: S1: 2.4 S2: 4.96 S3: 1.45 S4: 1.54 S5: 1.22 S6: 1.19 Third hair sample: S1: 2.8 S2: 1.69 S3: 1.61 S4: 1.59 S5: 1.94 S6: 3.95 S7: 1.64 S8: 1.39 S9: 1.28
	26‐year‐old female, who consumed a few alcoholic drinks at a party, after which she began to feel sleepy	First hair sample, 5.9 cm (1 day after the incident) Second hair sample, 7 cm (1 month after the incident) 5‐mm segments	First hair sample: S1: 3.01 S2: 2.04 S3: 2.16 S4: 1.99 S5: 1.87 S6: 4.3 S7: 2.32 S8: 1.83 S9: 1.79 S10: 1.85 S11: 1.89 S12: 1.81 Second hair sample: S1: 3.11 S2: 6.41 S3: 2.24 S4: 1.95 S5: 1.79 S6: 1.88 S7: 1.8 S8: 3.85 S9: 2.3 S10: 1.89 S11: 1.48 S12: 1.57 S13: 1.61 S14: 1.59
	20‐year‐old male, occasional cocaine user, who reported that he had taken GHB in the past (approximately 3 months earlier)	3.6‐cm hair sample 5‐mm segments	S1: 4.54 S2: 3.15 S3: 3.29 S4: 3.45 S5: 4.19 S6: 3.95 S7: 3.21
Busardò et al. [32]	45‐year‐old man who had undergone sodium oxybate treatment for 6 months	10‐cm gray hair sample 1‐cm segments	S1: 9.23 S2: 8.48 S3: 7.35 S4: 8.42 S5: 7.75 S6: 8.06 S7: 2.82 S8: 1.24 S9: 0.95 S10: 0.89
Carfora et al. [23]	56‐year‐old female tourist who claimed that she was raped under the effect of drugs	22‐cm hair sample 1–3‐mm segments	S1: 0.8 S2–S12: 0.54–0.63
Fischer et al. [25]	51‐year‐old man who was found dead in his bed due to the oral ingestion of 1,4‐butanediol (1,4‐BD), a GHB metabolic precursor	1‐cm black hair sample	26.7
Goullé et al. [26]	35‐year‐old White man abusing GHB by oral route to promote muscle mass	2‐cm black hair sample	14.0
	29‐year‐old Black woman, who was raped under the influence of GHB	24‐cm black hair sample (7 days after the rape) 3‐mm segments	3 (proximal) segments: 3.1, 5.3, and 4.3 77 (distal) segments: 0.71 (median concentration)
Jamey et al. [18]	69‐year‐old man who was discovered dead	4–6‐cm gray hair sample	96.3
Kintz et al. [36]	43‐year‐old Caucasian man (known drug abuser) who was found unconscious and later pronounced dead	Pubic hair 8‐mm segments	S1: 25.0 S2: 22.6 S3: 19.4
Küting et al. [21]	40‐year‐old man who was found dead in bed	4.5‐cm hair sample 0.5‐cm segments	Unwashed hair: S1: 174 S2: 147 S3: 118 S4: 102 S5: 97.8 S6: 95.2 S7: 91.9 S8: 128 S9: 141 Washed hair: S1: 131 S2: 134 S3: 113 S4: 100 S5: 98.2 S6: 97.1 S7: 49.2 S8: 81.1 S9: 105
Mehling et al. [37]	6‐year‐old girl who died from a GHB overdose, which was administered by the minor's uncle to chemically subdue her in order to carry out sexual abuse	16‐cm hair sample 2‐cm segments for the first 4‐ and 1‐cm segments for the remaining hair length	S1: 40.0 S2: 20.0 S3: 30.0 S4: 25.0 S5: 20.0 S6: 20.0 S7: 15.0 S8: 5.0 S9–S13: 2.0–3.0
Paul et al. [13]	25‐year‐old man who admitted recreational use of GHB on one occasion	5‐cm hair sample 5‐mm segments	Endogenous levels: 0.11–0.3 Exogenous levels: S5: 4.5; S6: 3.81
	Male arrested for bodily harm following an assault at a party and stated that he had lost all memory from half‐way through the party	4‐cm hair sample 5‐mm segments	Endogenous levels: 0.1–0.19 Exogenous levels: 3.23
Rossi et al. [12]	24‐year‐old girl who suspected that she was raped under the effect of drugs	20‐cm hair sample 3‐cm segments for the first 12 cm (proximal) 2‐cm segments for the 8‐cm distal part	Proximal segments: S1: 5.0 S2: 4.0 S3: 3.0 S4: 4.0 Distal segments: 1.0 (median concentration)
Shi et al. [17]	20‐year‐old girl who reported a sexual assault case	6‐cm hair sample (1 month after the incident) 1‐cm segments	S1, S3–S6: < 2.0 S2: 4.31
Van Elsué et al. [19]	10 GHB‐dependent patients (3 males and 7 females)	3‐cm proximal segment	6.3–239.6
Wang et al. [33]	8 drug‐rape cases	3‐mm segments	< LOQ(0.32)–2.2
	A couple who admitted GHB abuse and who applied for treatment (male and female)	Male (dark brown): 3‐cm hair sample 3‐mm segments Female (light brown and thin): 14.5‐cm hair sample 5‐mm to 2.5‐cm segments	Male: 461–594 Female: 27–159

## 5 Conclusions

The developed method allows the detection and quantification of GHB by UPLC‐MS/MS. The sample analysis is carried out with a small amount of hair (10 mg), in a relatively short period of time, approximately 1.5 h, and avoiding the use of organic solvents. The validation process showed satisfactory results for all the studied parameters and, therefore, demonstrated that the method is exact, precise, selective, and sensitive. The method proved to be linear in a concentration range of 0.5–50 ng/mg, allowing us to make a distinction between GHB endogenous levels and exogenous administration. Moreover, it was applied to the analysis of hair samples from 26 different individuals, establishing an endogenous concentration range between < LOQ and 6.25 ng/mg and allowing us to determine exogenous exposure by using the approach based on the ratio calculation between different segments of the same individual. In addition, a review of endogenous and exogenous GHB hair concentrations reported in the literature is also summarized.
